# What are the perceptions and experiences of adults using mobile applications for self-management in diabetes? A systematic review

**DOI:** 10.1136/bmjopen-2024-086671

**Published:** 2025-01-20

**Authors:** Kalthum Patail, Hui Hsuan Pai, Geva Greenfield, Benedict Hayhoe, Azeem Majeed, Ana Luisa Neves, Henock B Taddese

**Affiliations:** 1 Primary Care and Public Health, Imperial College London, London, UK; 2 Imperial College London, London, UK; 3 Department of Primary Care & Public Health, Imperial College London Faculty of Medicine, London, UK; 4 Imperial College London Faculty of Medicine, London, UK; 5 Primary Care, Imperial College London, London, UK; 6 Department of Primary Care and Public Health, School of Public Health, Imperial College London Faculty of Medicine, London, UK

**Keywords:** Patients, Self-Management, Review, eHealth, Cell Phone, DIABETES & ENDOCRINOLOGY

## Abstract

**Abstract:**

**Objectives:**

Diabetes mellitus is a chronic disease that entails significant burdens to patients, caregivers and society at large. While self-management behaviours like healthy eating and monitoring of blood glucose help to reduce the care burden, they are still perceived to be burdensome. Mobile applications have emerged as promising digital tools in helping patients to self-manage their conditions. We conducted systematic review to explore the perceptions and experiences of adults with types 1, 2 and gestational diabetes using mobile applications for self-management in diabetes.

**Design:**

A systematic review of published primary studies exploring perceptions and experiences of adults living with types 1, 2 and gestational diabetes who used/have used mobile applications for self-management. The study was reported following the best practice guidelines defined in the Preferred Reporting Items for Systematic Reviews and Meta-Analyses.

**Data sources:**

We searched for articles published from January 2007 to December 2023 via MEDLINE (Ovid), Embase (Elsevier), CINAHL (Ovid) and Scopus (Elsevier).

**Eligibility criteria:**

Primary qualitative studies that describe the perceptions and experiences of adults in using mobile applications in self-management of types 1, 2 and gestational diabetes published between January 2007 and December 2023.

**Data extraction and synthesis:**

Two independent reviewers screened identified papers for eligibility, extracted data using a pre-defined data extraction form and applied the Critical Appraisal Skills Program tool to appraise the quality of the included studies. Data were narratively synthesised, guided by the ‘unified theory of acceptance and use of technology (UTAUT)’ framework.

**Results:**

A total of 24 qualitative studies deploying interviews and surveys with open-ended questions were included in the review. We identified four major themes, which were mapped against the constructs of the ‘UTAUT’ framework: ‘performance expectancy’, ‘effort expectancy’, ‘social influence’ and ‘facilitating conditions’.

More than 50% of the studies described favourable app features relating to monitoring blood glucose, diet and exercise while also emphasising the importance of customising these to patient needs. About 40% of the studies described unfavourable aspects related to uploading of excessive information, monitoring device incompatibility, episodic app crashes and telephone handling issues.

**Conclusion:**

The review supports the favourable view of mobile applications as promising tools in helping patients with diabetes to self-manage. However, the challenges on the ease of use and non-customised features of the apps potentially hinder patients’ long-term engagement.

**STRENGTHS AND LIMITATIONS OF THIS STUDY**

- The review has included evidence from studies conducted in Asia, Europe and America with varying backgrounds: age, educational status, gender as well as diabetes types 1, 2 and gestational.
- The qualitative data generated through semi structured interviews, face-to-face, telephone or focus group discussions provided rich insights into patient experiences.
- Mixed method studies were excluded in the review, however, the amount of qualitative data from the identified mixed method studies was small and would not have added fresh insights.
- There is some degree of bias in the selection of participants as most of them were recruited from health centres and clinics, signifying that participants are likely to have positive health seeking behaviour and may already be receptive towards self-management.
- Most of the participants in our review had only been using the apps for less than 6 months; therefore, findings may not aid generalisations in relation to long term or sustainable use of apps.

## Introduction

 Globally, diabetes is one of the fastest growing chronic diseases in the twenty-first century alongside cardiovascular diseases and cancer. Type 1, type 2 and gestational diabetes are the different types of metabolic disorders that develop based on unique physiological changes. Common to all types of diabetes, the body manifests higher than normal blood glucose levels due to absence of or resistance to a hormone called insulin, signifying failure to efficiently use glucose for energy.^1^

The number of people living with diabetes is predicted to increase from 537 million in 2021 to 643 million in 2030 and is further expected to rise to 783 million by 2045.^1^ The disease has a debilitating effect on blood vessels and nerves, leading to death from cardiovascular diseases, vision impairment and disability from amputations as a result of diabetic foot ulcers.^1^ The increasing prevalence represents a substantial burden to global health expenditures, which are projected to rise from USD 1.3 trillion in 2015 to more than USD 2.1 trillion by 2045.^2^ Further aggravating the situation is the constraint on global medical workforce which does not grow in tandem with the diabetic population, leading to overburdened workforce.^3^ This all implies that the current system of care must go beyond solely treating the disease to equipping patients with skills to self-manage. However, self-management tasks which encompass blood glucose monitoring, physical activity and nutrition tracking, medication adherence and knowledge acquisition are perceived to be complex and overwhelming.^4^

### Use of technology in self-management

The WHO has recognised leveraging on mobile applications as the way forward in assisting patients to manage self-monitoring tasks.^5^ Diabetes self-management mobile applications consist of features such as enabling patients to record their health information in text or video formats. Based on this information, self-care activities can be tailored, and users are automatically reminded to perform them.^6^ According to data gathered in 2014 through health information trend surveys, mobile health app users found the apps beneficial in achieving their health goals, facilitating medical care decision-making and offering an opportunity to ask their physicians questions and for seeking second opinions.^7^ While mobile applications have demonstrated to be beneficial, the fast growth of innovations in their design has led to some confusion and difficulty in usage among patients.^8^

### The knowledge gap in users’ perceptions and experiences

A few studies have emphasised the need for user-based design to support individual patients’ needs and to enable maximum app acceptance and usability.^9 10^ In a diabetes app evaluation study done by Brzan *et al*,^7^ there were many applications that were unable to distinguish between type 1 and type 2 diabetes or even gestational diabetes resulting in confusion in the different meal, exercise and medication plans for the specific conditions. Another study that examined users’ preferences in a multinational setting revealed that patients have differing preferences based on the type of diabetes.^11^ For instance, most patients with type 2 diabetes felt a feature in an app that nudges a user to engage in positive behaviour would be helpful, such as geofencing a user when a binge eating habit is detected.^12^ On the other hand, patients with type 1 diabetes who were in their early 20s placed high importance on information on how to cope with self-monitoring tasks during the transition from teen to young adult and features that allow interfacing with social media platforms to share experiences.^12^ Despite the recognised need for user-centred research to improve the usability and feasibility of diabetes mobile apps, most existing systematic reviews focus on effectiveness of the applications in terms of blood glucose monitoring, technical feasibility and capturing data on patterns of usage.^9^,^11^ The aim of this study is to collate and synthesise evidence focusing on patients’ perceptions and experiences in using these apps.

## Methods

### Study design

The study was reported following the best practice guidelines defined in the Preferred Reporting Items for Systematic Reviews and Meta-Analyses (PRISMA) to ensure our review meets the standard of producing reproducible and verifiable studies.^13^ Only qualitative studies using methods such as in-depth interviews, focus groups and surveys (open ended questions) were included as these methodologies are optimal to capture users’ beliefs, attitudes and experiences.

### Search strategy

A comprehensive search was conducted in MEDLINE (Ovid), Embase (Elsevier), CINAHL (Ovid) and Scopus (Elsevier) for relevant articles published from January 2007 to March 2023 (online supplemental appendix 1). The year 2007 was selected because that year marked the birth of smart phones that incorporate videos, cameras and applications.^14^ We used subject headings, truncation and keywords related to mobile applications, mobile health application, cell phone, smart phone, mobile health technology, smart phone application, remote monitoring, insulin and non-insulin dependent, type 1 and type 2 diabetes, gestational diabetes, self-management, self-care and self-efficacy to retrieve relevant articles. Online supplemental appendix 1 lists the full search strategy deployed in these databases.

### Eligibility criteria

We included studies that consist of adults aged 18 and above who have type 1, type 2 or gestational diabetes and who expressed their thoughts, perceptions and experiences of using mobile applications for self-management tasks such as blood glucose monitoring, physical activity and weight monitoring, diet tracking, medication adherence and knowledge acquisition. We only included studies that use mobile applications exclusively or mobile applications in conjunction with text messages and wearable devices to communicate with healthcare providers. We considered published studies in all languages, not just limited to English publications only. On the other hand, studies discussing mobile app users with mental health conditions such as dementia or other psychological impairment were excluded. Emailing, text messages and any technology used solely as a means of communication with the healthcare providers to manage the condition were not considered because they provided limited capabilities in self-management functionalities. Please see Eligibility Criteria table below (table 1).

**Table 1:** Eligibility criteria

	Inclusion criteria	Exclusion criteria
Population	Adults 18 years and above with diabetes type 1, type 2 or gestational diabetes. Diabetes with no specification types or with comorbidities	Paediatrics, adolescence diabetes and psychologically impaired patients
Intervention	Mobile phone applications in conjunction with text messages and wearable devices. Tablets with mobile applications	Tele medicine, video conferencing, emailing and text messages exclusive for communications with health service providers
Comparator	Not applicable	Not applicable
Outcomes	Experiences, perceptions, thoughts about the use of mobile applications for blood glucose monitoring, diet, physical activities, medication adherence and education	Perceptions of care givers, healthcare providers and family members
Study design	Publications from 2007 to 2023. Studies consisting qualitative methods such as in-depth interviews, focus groups and surveys	RCTs, cross-sectional, cohort, case controls, cost-effectiveness studies

### Screening and data extraction

The eligibility criteria guided the two independent reviewers, KP and PHH, during title, abstract and text screening process. Any disagreements on the studies selected were resolved by a third independent assessor, GG. Information from the identified studies including publication type, name of author, intervention, study type, participants and outcome were extracted and captured in a standardised data extraction form.

The Critical Appraisal Skills Program (CASP) tool for assessing qualitative studies was used to critically appraise the studies.^15^ In terms of theoretical evidence, the Unified Theory of Acceptance and Use of Technology (UTAUT) was used to underpin our analysis, providing a focus on factors that drive or deter the patients from using mobile applications.^16^ The four major constructs within the framework that are applied to understand patients’ behavioural intention are ‘performance expectancy’, ‘effort expectancy’, ‘social influence’ and ‘facilitating conditions’. Venkatesh *et al*^16^ explained ‘performance expectancy’ as patients’ belief in the application as a self-management tool, ‘ease of use’ as how easy or difficult they find using the app, ‘social influence’ as how much weight friends and relatives can exert on the use of app and ‘facilitating conditions’ as factors that motivate or incentivise the adoption of apps. However, the constructs do not promise a linear prediction of patients’ behaviour as age, gender and experience are some confounding factors to be taken into consideration^16^

### Ethical consideration

In recent years, systematic reviews have become methodologically inclusive of different types of research, and ethical consideration has become more relevant for systematic reviews. We considered key ethical issues for study inclusion such as indications of informed consent from participants, sufficient details on how the research is explained to participants, attention on conflict-of-interest statements as well as any approvals sought from ethics committee to gather the primary data.

## Results

### Identification of studies

**Figure 1:**
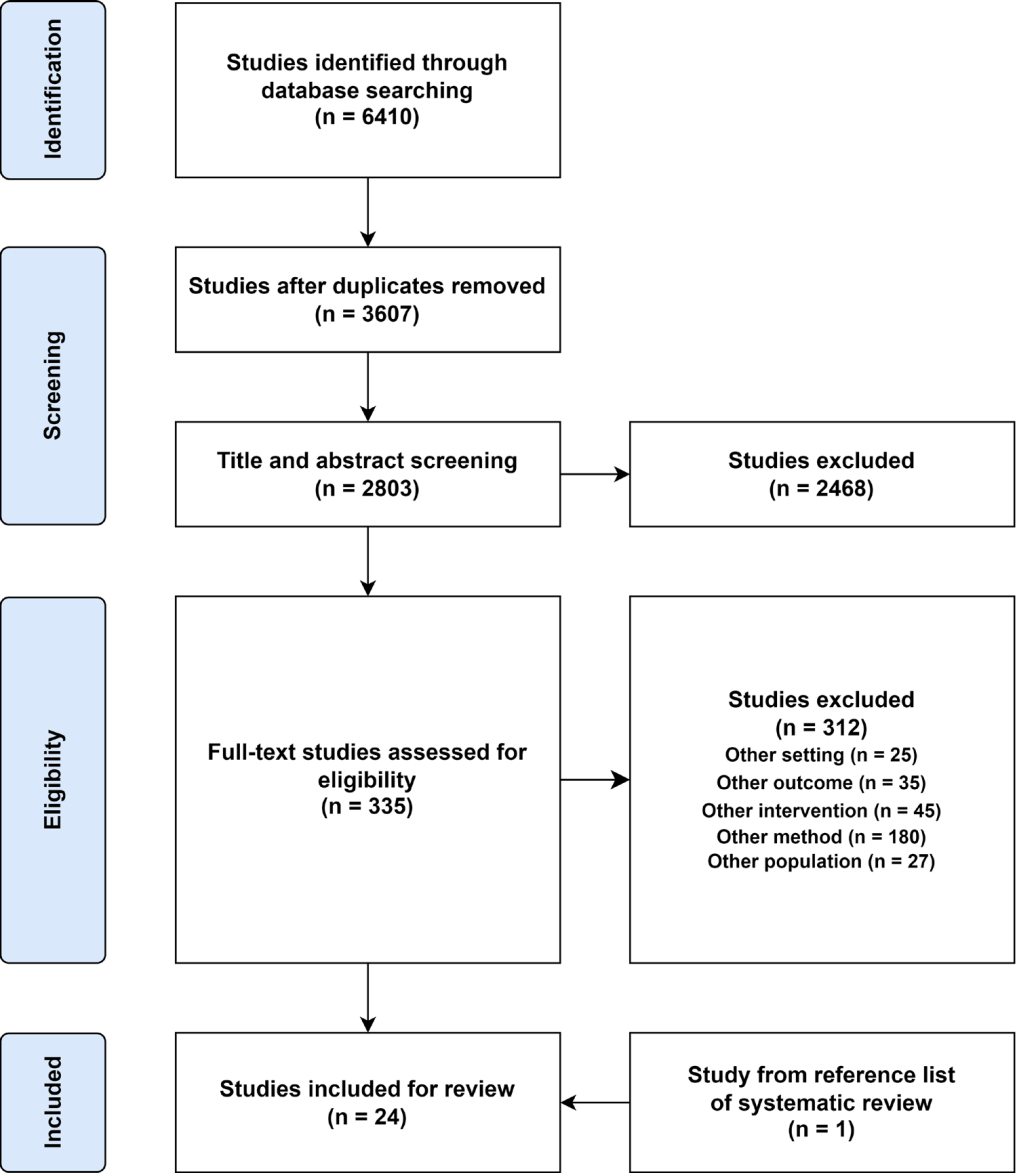
Preferred Reporting Items for Systematic Reviews and Meta-Analyses flowchart of Identified studies.

A total of 6410 studies were retrieved from MEDLINE (Ovid), EMBASE (Elsevier), CINAHL (Ovid) and SCOPUS (Elsevier). Further to removal of duplicates and title, abstract and full-text screening, 23 studies were deemed eligible as they met the inclusion criteria (figure 1). There were eight studies that were published in non-English languages: five in Spanish and three in Mandarin publications. The abstracts of these studies were translated by the native speakers of the respective languages. However. these studies were excluded because they did not meet the inclusion criteria. The main reasons for exclusion of studies include outcomes focused on healthcare personnel’s (HCP) perceptions, quantitative study designs, tele-monitoring with web-based blood glucose monitoring tracking systems and focus on paediatric populations. We further retrieved one study from a reference list search of a systematic review which made up a total of 24 included studies in the end. Please see PRISMA flow chart below (figure 1).

### Characteristics of studies

A summary of the characteristics of identified studies is provided in online supplemental appendix 2. The review brings together publications from March 2018 to April 2023, and the research in these studies spans across Asian, African and Western countries. Four studies were conducted in Asia, namely Singapore, Shanghai and Cambodia;^17^,^20^ two studies from Australia;^21 22^ five studies from Europe;^23^,^27^ two studies from the UK;^28 29^ eight studies from the USA;^30^,^37^ one study from Canada;^38^ one study from Central Africa;^39^ and one study reporting research done in both Singapore and Germany.^40^ In all but three studies, qualitative data were collected via face to face interviews. In just two studies, interviews were done over the telephone^34 38^ and in one by electronic survey with open-ended questions.^32^

### Characteristics of participants

The studies involved a wide range of diabetes conditions including type 1,^22 24 28 35^ type 2^18^,^2023 27 29^ and expectant mothers with type 2 and gestational diabetes;^21 36^ studies involving both type 1 and type 2 diabetes;^32 33 40^ one study with unspecified diabetes and type 2 diabetes plus hypertension;^17^ and one study with type 1, type 2 and a hybrid of type 1 and 2 diabetes.^25^ The age group of participants ranges between 23 and 81 years with 70% aged 45 and above. Most of the studies involved participants with minimum secondary or technical education. About seven studies involve participants who are students, employed adults with low to high social income as well as unemployed ones.

Four studies consist of participants with a mix of experienced and non-experienced app users for self-management in diabetes.^18 21 24 28^ Fourteen studies recruited participants with app experience ranging from 30 days to 22 months.^1922 23 27 29^,^31 33^ Two studies consist of non-experienced app users.^25 36^ One other study has no mention on the experience of using the app.^17^ In three studies, participants were either given prototypes or were asked to download commercially available apps with single or comprehensive diabetes self-management functions at the point of interview or simply answering a written set of questions expressing their ‘dream’ features in an app supporting self-management in diabetes.^20 26 32^

### Summary of quality appraisal of studies

All the studies were appraised as per the CASP guidelines which include 10 quality-related assessment questions (online supplemental appendix 3).^15^ The quality of the findings was rated from fair to good as shown in the last column of online supplemental appendix 2. Three studies^32 34 38^ were rated ‘fair’, due to the minor limitations such as the way data have been collected, for example, online survey or telephone interviews, appropriateness of the research design or the relationship between researcher and participants not well described. The rest of the studies were rated ‘good’ due to little or no flaws on the qualitative methodology with clear aims, research design being deemed appropriate to the aim of the studies and use of open-ended questions and focus groups to gather data.

### Outcomes

**Figure 2:**
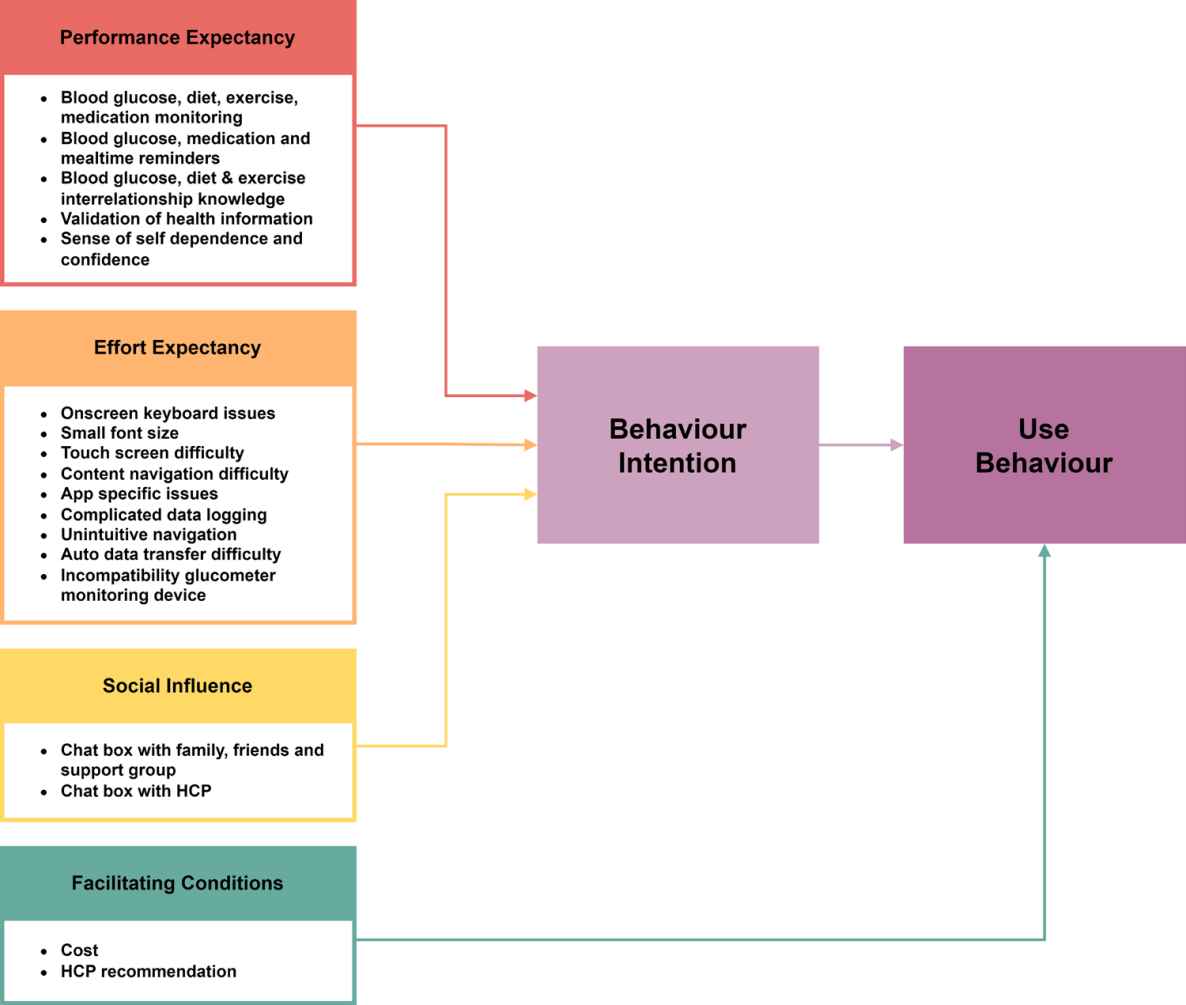
Summary of four major themes and subthemes mapped against the Unified Theory of Acceptance and Use of Technology. HCP, healthcare personnel.

The findings across the 24 studies were reviewed and synthesised to produce a summary table that captures participants experiences, perceptions and suggestions for improvements (online supplemental appendix 4). Findings were organised into themes and mapped across the UTAUT framework, which consist of four constructs namely ‘Performance Expectancy’, ‘Effort Expectancy’, ‘Social influence’ and ‘Facilitating Conditions’ (figure 2).

### Performance expectancy

‘Performance expectancy’ refers to the users’ opinion on the ability of the app in fulfilling the self-management tasks. Glucose monitoring, diet and exercise monitoring, reminder features and knowledge acquisitions were features that participants expected to help them reach their health goals^20^,^2527^ as shown in table 2. However, some of the participants expressed interest in apps with features tailoring to the different types of diabetes.^19 25 26 36^ The colourful graphs depicting interaction of diet and exercise with glucose levels were perceived to be educational and confidence enhancing.^1926 28 29 31^,^33 37 38 40^ Conversely, the visualisation of glucose and lifestyle trending charts which are meant to enhance accountability of individual actions were not favoured by participants from a rural community in the USA.^30^ Instead, the pen and paper method was preferred as it was perceived to be less intrusive and less accountable for own behaviour.^30^ As expressed by a 38-year-old female in the study by Peng *et al*:^30^

**Table 2:** App capability (performance expectancy)

App capability	Studies (%)	References
Colourful graphs depicting blood glucose, diet and exercise monitoring	20 (83%)	1921 25 27
Blood glucose levels, diet, exercise and medication interrelationship knowledge	17 (71%)	1921 22 27 29 31 33
Reminders on glucose checks, medication, medical appointment and mealtimes	8 (33%)	19 2228 35 38 39
Personalised features for types of diabetes	4 (17%)	19 25 26 36

*There’s that whole accountability piece. If I’m doing this. then I’m going to have to pay more attention. I want to eat my candy or my doughnut or whatever the case is*. (p.735)

In the same study, a 57-year-old male was satisfied with logbook which was perceived to be adequate.

Reminder features that support alerts on taking medication, checking blood glucose and doctors’ appointments were highly valued in 38% of the studies.^19^,^2228 35 38 39^ However, in two of the studies, tone of the reminder alerts were perceived to be annoying and generated stigmatisation.^24 32^ In addition, mealtime reminders were favoured better among the expectant mothers with gestational diabetes.^28^ This is possibly due to participants’ hectic working schedules and the emphasis of eating small frequent meals during gestation.^38^

Participants in over half of the studies valued the apps as source of information on the effect of diet, exercise and medication on the glucose levels.^1921^,^25 27^The knowledge acquired became beneficial during clinic visits, generating meaningful conversations with physicians which facilitated shared clinical decision making.^41 42^ While participants appreciated health education provided by the apps, there were also questions related to the validity and accuracy of the information provided.^21 23^ The participants were uncertain whether the information provided was validated by medical doctors.^21 23^

### Effort expectancy

The ‘ease of use’ factor played a key role in increasing users’ acceptance of the apps. Two main categories were identified as barriers to the use of apps, namely, onscreen keyboard issues and app specific issues, as shown in table 3. About 40% of the studies report participants facing challenges such as frequent crashing of apps, slow downloading of information, interoperability issues such as incompatible monitoring device with app and Bluetooth connectivity issues.^18 20 21 25 27 29 31 34 38 40^ In addition to app-specific technical issues, the older age group of participants also faced phone-related issues such as small font size, touch screen difficulties, switching between numbers and letters, and content navigation difficulties. The latter challenges were all related to participants aged 55 and above who were lacking in phone and computer literacy or were less experienced using smart phones.^17 18 21 25 30^

**Table 3:** ‘Ease of use’ (effort expectancy) themes and subthemes

User-friendliness issues	Studies (%)	References
Onscreen key board issues Small font size Touch screen difficulty Content navigation difficulty	5 (21%)	17 18 21 25 30
App technical issues Complicated data logging Unintuitive navigation Lack of data auto transfer to HCP Incompatible glucometer reading device Apps crashing Lack of automated uploading of information	10 (42%)	19 2125 27 29 31 34 38 40

HCPhealthcare personnel

Another common barrier in using the app perceived by many participants in many of the identified studies related to the requirement to upload excessive information. The lack of automated capability for uploading information had made the task of providing their health data to be overwhelming and time-consuming.^19 25 27 29 38 40^

### Social influence

In relation to social influence, eight studies illustrated the importance of family, support group and friends as well as HCP support for the acceptance and optimal use of the apps.^19 20 23 24 26 36 39 40^ The participants cited exchange of ideas and technical support to be the main facilitating factors for effective use of the app, particularly highlighting the importance of chat boxes to exchange ideas with other people living with diabetes. As a 47-year-old Chinese participant from integrated, multidisciplinary centre for diabetes and metabolism in Singapore expressed:^19^

*Peer support is a source of motivation. You can use the app to chat “so how do you maintain your sugar level? How do you go about doing it?”So, it’s not just one-way system. I mean if the app is just technical, it’s dead and the information you get from it is very fixed. But when you chat with a fellow diabeticpatient, you can learn and support each other.*

In some of the studies, the participants expressed interest in apps providing real time conversations with HCP so as to facilitate any corrective actions on glucose levels.^36^ For instance, in one of the studies,^36^ in a setting of pregnant women with diabetes using mHealth applications, real-time communications with health providers were appealing because they were unfamiliar with the disease and struggled to keep the glucose levels consistent. One of the participants from Northwestern Memorial Hospital in Chicago who favoured the sharing of glucose readings in real time with HCP commented:^36^

*They can reach out to you and be like…let’s schedule a visit a bit earlier so you can come in and I can help get you back on track…*”

### Facilitating conditions

Support for costs and medical endorsement of the apps were perceived to be beneficial in facilitating the uptake of the apps.^18 21 23 25 27 30 40^ It was suggested that the costs could be managed via health insurance schemes or subsidy from government. In addition, medical endorsement of the apps would help in strengthening trust for the app. Three studies indicated that participants would not use the apps unless recommended by their attending physicians.^23 30 40^

## Discussion

In general, patients were positive towards embracing technology in assisting them with self-monitoring tasks in diabetes management. The visual presentations illustrating associations between glucose levels with diet and exercise were the most favoured features; participants were also interested in personalising some of these features according to types of diabetes. Similar positive sentiments were echoed in findings from other authors discussing use of mobile apps in hypertension and cardiovascular diseases.^43^,^46^ For example, in cardiovascular-related apps, weight, water, blood pressure and diet trackers were highly valued.^44^ Similarly, in a systematic review exploring use of mobile apps in self-management in hypertension, the visual presentation of blood pressure readings in comparison to their water and salt intake was said to provide users with a better understanding of their conditions.^46^

The option to customise some of the features in the app was also favoured, which is a finding that is mirrored in studies exploring use of mobile applications among heart disease, hypertension and diabetes population.^42 44 46^ Complexity of the disease, ageing process and comorbidity explained the need for customisation.^47^ In the context of diabetes, the importance of customising some of the app functions according to diabetes type is vital. According to a study by Brzan *et al*^7^ on evaluation of diabetes health apps, the need for individualised functionality is preferable because the monitoring of blood glucose frequencies and medication therapy differs across type 1 and type 2 diabetes. Most patients with type 2 diabetes do not monitor blood glucose daily, and most of them receive oral therapy.^48^ On the other hand, type 1 diabetes patients are on insulin therapy, and there is a need for daily blood glucose monitoring and access to insulin bolus calculator.^41 49^ The sensitivity and complexity of insulin dose titration explained the highly sought after feature of apps: graphs depicting relationship between carbohydrate intake, insulin dose and blood glucose level.^50^

Reminder features, especially for medication and doctor appointments, were perceived to play an integral role in attaining patients’ health goals. This sentiment was also similarly expressed in other studies that represented the use of mobile apps in the management of chronic diseases like cardiovascular diseases, particularly in the older population.^44 51^ App developers should consider customising notification alerts according to individual preferences, for example, a customised ringtone and the ability to turn off reminder at specific times. According to a study that explored patients’ preferences for healthcare reminders and notification alerts in chronic disease management apps, reminders which were not need-specific ran the risk of hindering users from achieving their health goals.^52^ Motivational reminders such as socially supportive reminders that explain the importance of performing the task could be a way to address this issue.^52^

Our review also reflects the importance of HCP endorsement as a driving force in patients adopting the app. However, the apps that patients preferred were not always accepted by clinicians. This is due to massive disparities in clinical approval processes from country to country.^53 54^ This further highlights the need for close collaboration among clinicians, patients and regulators during the design phase.

In our review, we show that if apps are not user friendly, beneficial features perceived by patients would be short-lived, and there would be tendency to disengage.^42^ Age-related onscreen keyboard issues and app-specific issues faced by all ages, like episodic app crashes, poor connectivity and incompatibility of monitoring device were common concerns shared by patients using chronic disease apps.^55^,^57^ Still, an expert-led usability test of general diabetes apps for 50 years and older demonstrated promising result of moderate to good usability for 50–60 years of age.^58^ These apps offered smaller range of functions which were valued and seen to be less confusing than the multi-functional apps that performed the least in usability.^58^ In order to help seniors cope better, app developers should consider training or incorporating welcoming wizards of how to get started in using the apps.^57 59^ Failure to look into designing apps for the older age groups would lead to inability to reach a major target of a key demographic group that is most affected by diabetes.^60^

Excessive uploading of information was perceived to be time-consuming and frustrating; these were not only expressed in our review but also cited by other studies.^45 61^ Technical difficulties, lack of time and discipline to upload the excessive information were all common concerns echoed in these studies. Automated loading of health data was preferred as less time and effort were needed to input the requisite information. While automated data loading represents simplicity in the process of documenting, unfortunately, it could also pose some technical challenges in terms of frequent app crashes, which in turn resulted in loss of patients’ health data.^62^ In one study that reported crowd-sourced negative user review comments of 47 mobile apps from the Google play store, extremely slow speed of data uploading, app crashes resulting in loss of record and incompatibility of monitoring devices with app constituted the major aspect of technical barriers.^63^

### Future studies and policy implications

Given that more than 90% of these identified studies consist of participants using the apps for less than 6 months, it is difficult to predict the experience of longer usage of the apps in diabetes management and the role it will play in the entire healthcare system. The studies also revealed that ‘peer support’ function facilitating exchange of ideas in managing diabetes is a source of motivation for patients. Hence, future studies that capture long-term usage of mobile apps incorporating personal AI in providing an interactive support that assists patients with self-management in the disease are recommended. Through this review, we highlighted the need for medical endorsement to increase credibility and trust in using the apps. Therefore, we recommend HCPs to be included in future studies to gauge views on the apps.

As observed in our review, patients were receptive towards embracing technology towards helping them to attain health goals. Policy actors, namely health ministry and regulators, should work towards providing necessary support like training and guidance to the HCPs. With a structured support, they would be more inclined towards incorporating mobile app in the management of diabetes, thus further enhancing the confidence in patients using the apps.

### Strengths and limitations

The review provides an in-depth understanding of patients’ experiences, views and barriers in using mobile applications for self-management of diabetes. The review has included studies conducted in Asia, Europe and America with participants of different backgrounds and cultures which add to the richness of the data gathered. The varying age, educational status, gender and inclusion of diabetes types 1, 2 and gestational in these studies highlight the importance of tailoring to individual needs. Even though the sample sizes were small across most studies, the qualitative method of employing semistructured interviews, face to face, telephone or focus group discussions provided rich insights into patient experiences.

The review findings are to be taken with some considerations due to some limitations. We limited our review to qualitative studies which conducted semistructured interviews that could provide rich data, excluding the mixed method studies. However, the amount of data from the identified mixed method studies was small and would not have added fresh insights. In addition, there is some degree of bias in selecting participants as most of them were recruited from health centres and clinics who were already receptive towards self-management at baseline. Moreover, most of the participants in our review were using the apps for less than 6 months; therefore, findings may not aid generalisations in relation to long term or sustainable use of apps.

### Conclusion

Patients were receptive towards adopting mobile applications and felt more confident and self-reliant in the tracking of their glucose, diet, medication and exercise. However, these positive sentiments would be short lived if technical difficulties and non-customised features faced by the population were not addressed. If the ease of use issues and specific needs for the different diabetes types are unmet, patients may not engage sustainably with the app. It is crucial to support HCPs by incorporating digital health into medical curriculum and increasing tripartite collaboration among the government, tech companies and health institutions for an improved regulatory digital health framework. This will potentially enhance the inclusion of mobile health apps in the clinical care pathway.

## supplementary material

10.1136/bmjopen-2024-086671 online supplemental file 1

## Data Availability

No data are available.
