# Supplementary material for: What are the perceptions and experiences of adults using mobile applications for self-management in diabetes? A systematic review
Source: BMJ Open. 2025 Jan 20;15(1):e086671. doi: 10.1136/bmjopen-2024-086671 (PMC11751966; doi:10.1136/bmjopen-2024-086671)
Supplement: online supplemental file 1 [file bmjopen-15-1-s001.docx]

# Appendices

## Appendix 1 – Search Strategy Table

| MEDLINE | |
| --- | --- |
| 1 | mobile app*.mp. [mp=title, abstract, heading word, drug trade name, original title, device manufacturer, drug manufacturer,  device trade name, keyword heading word, floating subheading word, candidate term word] |
| 2 | cell phone*.mp. [mp=title, abstract, heading word, drug trade name, original title, device manufacturer, drug manufacturer,  device trade name, keyword heading word, floating subheading word, candidate term word] |
| 3 | health app*.mp. [mp=title, abstract, heading word, drug trade name, original title, device manufacturer, drug manufacturer,  device trade name, keyword heading word, floating subheading word, candidate term word] |
| 4 | (mobile adj2 application).mp. [mp=title, abstract, heading word, drug trade name, original title, device manufacturer, drug  manufacturer, device trade name, keyword heading word, floating subheading word, candidate term word] |
| 5 | Smart phone*.mp. [mp=title, abstract, heading word, drug trade name, original title, device manufacturer, drug manufacturer,  device trade name, keyword heading word, floating subheading word, candidate term word] |
| 6 | (Smart adj2 application).mp. [mp=title, abstract, heading word, drug trade name, original title, device manufacturer, drug  manufacturer, device trade name, keyword heading word, floating subheading word, candidate term word] |
| 7 | (mobile adj2 technology).mp. [mp=title, abstract, heading word, drug trade name, original title, device manufacturer, drug  manufacturer, device trade name, keyword heading word, floating subheading word, candidate term word] |
| 8 | mobile monitoring.mp. [mp=title, abstract, heading word, drug trade name, original title, device manufacturer, drug  manufacturer, device trade name, keyword heading word, floating subheading word, candidate term word] |
| 9 | remote monitoring.mp. [mp=title, abstract, heading word, drug trade name, original title, device manufacturer, drug  manufacturer, device trade name, keyword heading word, floating subheading word, candidate term word] |
| 10 | diab*.mp. [mp=title, abstract, heading word, drug trade name, original title, device manufacturer, drug manufacturer, device  trade name, keyword heading word, floating subheading word, candidate term word] |
| 11 | exp Diabetes Mellitus, Type 2/ |
| 12 | exp Diabetes Mellitus, Type 1/ |
| 13 | exp Diabetes Mellitus/ |
| 14 | insulin dependent.mp. |
| 15 | non insulin dependent.mp. [mp=title, abstract, heading word, drug trade name, original title, device manufacturer, drug  manufacturer, device trade name, keyword heading word, floating subheading word, candidate term word] |
| 16 | Gestational diab$.mp. [mp=title, abstract, heading word, drug trade name, original title, device manufacturer, drug  manufacturer, device trade name, keyword heading word, floating subheading word, candidate term word] |
| 17 | self man*.mp. [mp=title, abstract, heading word, drug trade name, original title, device manufacturer, drug manufacturer,  device trade name, keyword heading word, floating subheading word, candidate term word] |
| 18 | selfcare.mp. [mp=title, abstract, heading word, drug trade name, original title, device manufacturer, drug manufacturer,  device trade name, keyword heading word, floating subheading word, candidate term word] |
| 19 | self efficacy.mp. [mp=title, abstract, heading word, drug trade name, original title, device manufacturer, drug manufacturer,  device trade name, keyword heading word, floating subheading word, candidate term word] |
| 20 | exp self efficacy/ |
| 21 | 1 or 2 or 3 or 4 or 5 or 6 or 7 or 8 or 9 |
| 22 | 10 or 11 or 12 or 13 or 14 or 15 |
| 23 | 17 or 18 or 19 or 20 |
| 24 | 21 and 22 and 23 |
| 25 | 21 and 22 |
| 26 | (self- monitor* or self monitor*).mp. [mp=title, abstract, heading word, drug trade name, original title, device manufacturer,  drug manufacturer, device trade name, keyword heading word, floating subheading word, candidate term word] |
| 27 | 17 or 18 or 19 or 20 or 26 |
| 28 | 21 and 22 and 27 |

| EMBASE | |
| --- | --- |
| 1 | mobile application*.mp. [mp=title, abstract, heading word, drug trade name, original title, device manufacturer, drug  manufacturer, device trade name, keyword heading word, floating subheading word, candidate term word] |
| 2 | exp mobile application/ |
| 3 | (mobile adj2 application*).mp. [mp=title, abstract, heading word, drug trade name, original title, device manufacturer, drug  manufacturer, device trade name, keyword heading word, floating subheading word, candidate term word] |
| 4 | cell phone*.mp. [mp=title, abstract, heading word, drug trade name, original title, device manufacturer, drug manufacturer,  device trade name, keyword heading word, floating subheading word, candidate term word] |
| 5 | cell phone*.mp. |
| 6 | Smartphone*.mp. [mp=title, abstract, heading word, drug trade name, original title, device manufacturer, drug manufacturer,  device trade name, keyword heading word, floating subheading word, candidate term word] |
| 7 | exp smartphone/ |
| 8 | (mobile adj2 technology).mp. [mp=title, abstract, heading word, drug trade name, original title, device manufacturer, drug  manufacturer, device trade name, keyword heading word, floating subheading word, candidate term word] |
| 9 | mobile technology*.mp. [mp=title, abstract, heading word, drug trade name, original title, device manufacturer, drug  manufacturer, device trade name, keyword heading word, floating subheading word, candidate term word] |
| 10 | exp mobile phone/ |
| 11 | Smartphone application*.mp. [mp=title, abstract, heading word, drug trade name, original title, device manufacturer, drug  manufacturer, device trade name, keyword heading word, floating subheading word, candidate term word] |
| 12 | smartphone/ |
| 13 | remote monitoring*.mp. [mp=title, abstract, heading word, drug trade name, original title, device manufacturer, drug  manufacturer, device trade name, keyword heading word, floating subheading word, candidate term word] |
| 14 | remote monitoring*.mp. |
| 15 | mobile monitoring*.mp. [mp=title, abstract, heading word, drug trade name, original title, device manufacturer, drug  manufacturer, device trade name, keyword heading word, floating subheading word, candidate term word] |
| 16 | mobile monitoring*.mp. |
| 17 | mHealth.mp. [mp=title, abstract, heading word, drug trade name, original title, device manufacturer, drug manufacturer,  device trade name, keyword heading word, floating subheading word, candidate term word] |
| 18 | mHealth.mp. |
| 19 | diab$.mp. [mp=title, abstract, heading word, drug trade name, original title, device manufacturer, drug manufacturer, device  trade name, keyword heading word, floating subheading word, candidate term word] |
| 20 | exp diabetes mellitus/ |
| 21 | insulin dependent diab$.mp. [mp=title, abstract, heading word, drug trade name, original title, device manufacturer, drug  manufacturer, device trade name, keyword heading word, floating subheading word, candidate term word] |
| 22 | exp non insulin dependent diabetes mellitus/ |
| 23 | gestational diab$.mp. [mp=title, abstract, heading word, drug trade name, original title, device manufacturer, drug  manufacturer, device trade name, keyword heading word, floating subheading word, candidate term word] |
| 24 | exp pregnancy diabetes mellitus/ |
| 25 | self management.mp. [mp=title, abstract, heading word, drug trade name, original title, device manufacturer, drug  manufacturer, device trade name, keyword heading word, floating subheading word, candidate term word] |
| 26 | exp self care/ |
| 27 | self care.mp. [mp=title, abstract, heading word, drug trade name, original title, device manufacturer, drug manufacturer,  device trade name, keyword heading word, floating subheading word, candidate term word] |
| 28 | self efficacy.mp. [mp=title, abstract, heading word, drug trade name, original title, device manufacturer, drug manufacturer,  device trade name, keyword heading word, floating subheading word, candidate term word] |
| 29 | self monitor*.mp. [mp=title, abstract, heading word, drug trade name, original title, device manufacturer, drug manufacturer,  device trade name, keyword heading word, floating subheading word, candidate term word] |
| 30 | exp self monitoring/ |
| 31 | 1 or 2 or 3 or 4 or 5 or 6 or 7 or 8 or 9 or 10 or 11 or 12 or 13 or 14 or 15 or 16 or 17 or 18 |
| 32 | 19 or 20 or 21 or 22 or 23 or 24 |
| 33 | 25 or 26 or 27 or 28 or 29 or 30 |
| 34 | 31 and 32 and 33 |

| CINAHL | | | | | |
| --- | --- | --- | --- | --- | --- |
| # | Query | Limiters /Expanders | Last Run Via | Results | Action |
| S26 | S23 AND S24 AND S25 | Limiters - Date Published: 20070101-20231231 Expanders - Apply equivalent subjects Search modes - Boolean/Phrase | Interface - EBSCOhost Research Databases Search Screen - Basic Search Database - CINAHL | 646 | [Edit](javascript:__doPostBack('ctl00$ctl00$MainContentArea$MainContentArea$editControl$printHistory$HistoryRepeater$ctl00$linkEditSearch',''))S26 |
| S25 | S1 OR S2 OR S5 OR S6 OR S7 OR S8 OR S9 | Expanders - Apply equivalent subjects Search modes - Boolean/Phrase | Interface - EBSCOhost Research Databases Search Screen - Basic Search Database - CINAHL | 209,721 | [Edit](javascript:__doPostBack('ctl00$ctl00$MainContentArea$MainContentArea$editControl$printHistory$HistoryRepeater$ctl01$linkEditSearch',''))S25 |
| S24 | S18 OR S19 OR S20 OR S21 OR S22 | Expanders - Apply equivalent subjects Search modes - Boolean/Phrase | Interface - EBSCOhost Research Databases Search Screen - Basic Search Database - CINAHL | 113,224 | [Edit](javascript:__doPostBack('ctl00$ctl00$MainContentArea$MainContentArea$editControl$printHistory$HistoryRepeater$ctl02$linkEditSearch',''))S24 |
| S23 | S3 OR S4 OR S10 OR S11 OR S12 OR S13 OR S14 OR S15 OR S16 OR S17 | Expanders - Apply equivalent subjects Search modes - Boolean/Phrase | Interface - EBSCOhost Research Databases Search Screen - Basic Search Database - CINAHL | 55,665 | [Edit](javascript:__doPostBack('ctl00$ctl00$MainContentArea$MainContentArea$editControl$printHistory$HistoryRepeater$ctl03$linkEditSearch',''))S23 |
| S22 | lifestyle improvement | Expanders - Apply equivalent subjects Search modes - Boolean/Phrase | Interface - EBSCOhost Research Databases Search Screen - Basic Search Database - CINAHL | 551 | [Edit](javascript:__doPostBack('ctl00$ctl00$MainContentArea$MainContentArea$editControl$printHistory$HistoryRepeater$ctl04$linkEditSearch',''))S22 |
| S21 | self monitor? | Expanders - Apply equivalent subjects Search modes - Boolean/Phrase | Interface - EBSCOhost Research Databases Search Screen - Basic Search Database - CINAHL | 640 | [Edit](javascript:__doPostBack('ctl00$ctl00$MainContentArea$MainContentArea$editControl$printHistory$HistoryRepeater$ctl05$linkEditSearch',''))S21 |
| S20 | self efficacy | Expanders - Apply equivalent subjects Search modes - Boolean/Phrase | Interface - EBSCOhost Research Databases Search Screen - Basic Search Database - CINAHL | 36,334 | [Edit](javascript:__doPostBack('ctl00$ctl00$MainContentArea$MainContentArea$editControl$printHistory$HistoryRepeater$ctl06$linkEditSearch',''))S20 |
| S19 | self care | Expanders - Apply equivalent subjects Search modes - Boolean/Phrase | Interface - EBSCOhost Research Databases Search Screen - Basic Search Database - CINAHL | 69,741 | [Edit](javascript:__doPostBack('ctl00$ctl00$MainContentArea$MainContentArea$editControl$printHistory$HistoryRepeater$ctl07$linkEditSearch',''))S19 |
| S18 | self management | Expanders - Apply equivalent subjects Search modes - Boolean/Phrase | Interface - EBSCOhost Research Databases Search Screen - Basic Search Database - CINAHL | 38,452 | [Edit](javascript:__doPostBack('ctl00$ctl00$MainContentArea$MainContentArea$editControl$printHistory$HistoryRepeater$ctl08$linkEditSearch',''))S18 |
| S17 | remote monitoring | Expanders - Apply equivalent subjects Search modes - Boolean/Phrase | Interface - EBSCOhost Research Databases Search Screen - Basic Search Database - CINAHL | 2,188 | [Edit](javascript:__doPostBack('ctl00$ctl00$MainContentArea$MainContentArea$editControl$printHistory$HistoryRepeater$ctl09$linkEditSearch',''))S17 |
| S16 | mHealth | Expanders - Apply equivalent subjects Search modes - Boolean/Phrase | Interface - EBSCOhost Research Databases Search Screen - Basic Search Database - CINAHL | 21,606 | [Edit](javascript:__doPostBack('ctl00$ctl00$MainContentArea$MainContentArea$editControl$printHistory$HistoryRepeater$ctl10$linkEditSearch',''))S16 |
| S15 | mobile monitoring | Expanders - Apply equivalent subjects Search modes - Boolean/Phrase | Interface - EBSCOhost Research Databases Search Screen - Basic Search Database - CINAHL | 461 | [Edit](javascript:__doPostBack('ctl00$ctl00$MainContentArea$MainContentArea$editControl$printHistory$HistoryRepeater$ctl11$linkEditSearch',''))S15 |
| S14 | mobile technology | Expanders - Apply equivalent subjects Search modes - Boolean/Phrase | Interface - EBSCOhost Research Databases Search Screen - Basic Search Database - CINAHL | 3,109 | [Edit](javascript:__doPostBack('ctl00$ctl00$MainContentArea$MainContentArea$editControl$printHistory$HistoryRepeater$ctl12$linkEditSearch',''))S14 |
| S13 | mobile technology or mobile devices or cell phones or tablets | Expanders - Apply equivalent subjects Search modes - Boolean/Phrase | Interface - EBSCOhost Research Databases Search Screen - Basic Search Database - CINAHL | 22,225 | [Edit](javascript:__doPostBack('ctl00$ctl00$MainContentArea$MainContentArea$editControl$printHistory$HistoryRepeater$ctl13$linkEditSearch',''))S13 |
| S12 | smart*application | Expanders - Apply equivalent subjects Search modes - SmartText Searching | Interface - EBSCOhost Research Databases Search Screen - Basic Search Database - CINAHL | 664 | [Edit](javascript:__doPostBack('ctl00$ctl00$MainContentArea$MainContentArea$editControl$printHistory$HistoryRepeater$ctl14$linkEditSearch',''))S12 |
| S11 | smart phone* | Expanders - Apply equivalent subjects Search modes - Boolean/Phrase | Interface - EBSCOhost Research Databases Search Screen - Basic Search Database - CINAHL | 4,280 | [Edit](javascript:__doPostBack('ctl00$ctl00$MainContentArea$MainContentArea$editControl$printHistory$HistoryRepeater$ctl15$linkEditSearch',''))S11 |
| S10 | cell phone* | Expanders - Apply equivalent subjects Search modes - Boolean/Phrase | Interface - EBSCOhost Research Databases Search Screen - Basic Search Database - CINAHL | 2,936 | [Edit](javascript:__doPostBack('ctl00$ctl00$MainContentArea$MainContentArea$editControl$printHistory$HistoryRepeater$ctl16$linkEditSearch',''))S10 |
| S9 | non-insulin dependent diabetes | Expanders - Apply equivalent subjects Search modes - Boolean/Phrase | Interface - EBSCOhost Research Databases Search Screen - Basic Search Database - CINAHL | 55,788 | [Edit](javascript:__doPostBack('ctl00$ctl00$MainContentArea$MainContentArea$editControl$printHistory$HistoryRepeater$ctl17$linkEditSearch',''))S9 |
| S8 | insulin dependent diabetes | Expanders - Apply equivalent subjects Search modes - Boolean/Phrase | Interface - EBSCOhost Research Databases Search Screen - Basic Search Database - CINAHL | 74,387 | [Edit](javascript:__doPostBack('ctl00$ctl00$MainContentArea$MainContentArea$editControl$printHistory$HistoryRepeater$ctl18$linkEditSearch',''))S8 |
| S7 | gestational diabetes | Expanders - Apply equivalent subjects Search modes - Boolean/Phrase | Interface - EBSCOhost Research Databases Search Screen - Basic Search Database - CINAHL | 12,174 | [Edit](javascript:__doPostBack('ctl00$ctl00$MainContentArea$MainContentArea$editControl$printHistory$HistoryRepeater$ctl19$linkEditSearch',''))S7 |
| S6 | Type 2 diabetes | Expanders - Apply equivalent subjects Search modes - Boolean/Phrase | Interface - EBSCOhost Research Databases Search Screen - Basic Search Database - CINAHL | 88,924 | [Edit](javascript:__doPostBack('ctl00$ctl00$MainContentArea$MainContentArea$editControl$printHistory$HistoryRepeater$ctl20$linkEditSearch',''))S6 |
| S5 | Type 1 diabetes | Expanders - Apply equivalent subjects Search modes - Boolean/Phrase | Interface - EBSCOhost Research Databases Search Screen - Basic Search Database - CINAHL | 82,794 | [Edit](javascript:__doPostBack('ctl00$ctl00$MainContentArea$MainContentArea$editControl$printHistory$HistoryRepeater$ctl21$linkEditSearch',''))S5 |
| S4 | mobile*application | Expanders - Apply equivalent subjects Search modes - Boolean/Phrase | Interface - EBSCOhost Research Databases Search Screen - Basic Search Database - CINAHL | 7,698 | [Edit](javascript:__doPostBack('ctl00$ctl00$MainContentArea$MainContentArea$editControl$printHistory$HistoryRepeater$ctl22$linkEditSearch',''))S4 |
| S3 | mobile application* | Expanders - Apply equivalent subjects Search modes - Boolean/Phrase | Interface - EBSCOhost Research Databases Search Screen - Basic Search Database - CINAHL | 14,016 | [Edit](javascript:__doPostBack('ctl00$ctl00$MainContentArea$MainContentArea$editControl$printHistory$HistoryRepeater$ctl23$linkEditSearch',''))S3 |
| S2 | (MH "Diabetes Mellitus+") | Expanders - Apply equivalent subjects Search modes - Boolean/Phrase | Interface - EBSCOhost Research Databases Search Screen - Basic Search Database - CINAHL | 185,393 | [Edit](javascript:__doPostBack('ctl00$ctl00$MainContentArea$MainContentArea$editControl$printHistory$HistoryRepeater$ctl24$linkEditSearch',''))S2 |
| S1 | diab? | Expanders - Apply equivalent subjects Search modes - Boolean/Phrase | Interface - EBSCOhost Research Databases Search Screen - Basic Search Database - CINAHL | 696 | [Edit](javascript:__doPostBack('ctl00$ctl00$MainContentArea$MainContentArea$editControl$printHistory$HistoryRepeater$ctl25$linkEditSearch',''))S1 |

| SCOPUS | |
| --- | --- |
| 1 | Combination of 3 concepts  ( ( TITLE-ABS-KEY ( gestational  AND diab? ) )  OR  ( TITLE-ABS-KEY ( non  AND insulin  AND dependent  AND diab? ) )  OR  ( TITLE-ABS-KEY ( insulin  AND dependent  AND diab? ) )  OR  ( TITLE-ABS-KEY ( type  2  diab? ) )  OR  ( TITLE-ABS-KEY ( type  1  diab* ) )  OR  ( TITLE-ABS-KEY ( diabetes  AND mellitus ) )  OR  ( TITLE-ABS-KEY ( diab* ) ) )  AND  ( ( TITLE-ABS-KEY ( smartphone  AND application* ) )  OR  ( TITLE-ABS-KEY ( mobile  AND monitor? ) )  OR  ( TITLE-ABS-KEY ( mobile  AND technolog? ) )  OR  ( TITLE-ABS-KEY ( remote  AND monitoring* ) )  OR  ( TITLE-ABS-KEY ( smart  AND phone* ) )  OR  ( TITLE-ABS-KEY ( cell  AND phone* ) )  OR  ( TITLE-ABS-KEY ( mobile  AND application* ) ) )  AND  ( ( TITLE-ABS-KEY ( self  AND management ) )  OR  ( TITLE-ABS-KEY ( self  AND care ) )  OR  ( TITLE-ABS-KEY ( self  AND efficacy ) )  OR  ( TITLE-ABS-KEY ( lifestyle  AND improvement ) )  OR  ( TITLE-ABS-KEY ( self  AND monitor? ) ) )  AND  ( LIMIT-TO ( SUBJAREA ,  "MEDI" )  OR  LIMIT-TO ( SUBJAREA ,  "NURS" )  OR  LIMIT-TO ( SUBJAREA ,  "BIOC" )  OR  LIMIT-TO ( SUBJAREA ,  "HEAL" ) ) |

## Appendix 2 – Characteristics of identified studies

|  | Author | Study Method | Country | Population | Intervention | Outcome | Quality Appraisal Ranking |
| --- | --- | --- | --- | --- | --- | --- | --- |
| 1 | Bults M 2023 | Semi structured interviews by telephone focus group | Netherlands | 20 participants Type 2 diabetes Mean age 63 years | Freestyle Libre, MIGUIDE, selfcare and MYSUGR apps 4 months of using the app | Useful insights for healthy lifestyle; helped in glucose monitoring and diabetes control knowledge; app influences lifestyle; an eyeopener, inaccurate reading; visually appealing graphs; 8 hours BGM data transfer to phone; excessive food and physical activity data, assistance needed; inadequate social support; food habit challenge; financial reimbursement request. no issue; with security | Good |
| 2 | Kyoto M et al 2022 | Semi structured interview | Europe (Finland) | 10 women   Gestational diabetes (24 weeks) Mean age 33yrs | Prototype app presented to participants to browse | Self-tracking data; customisable habits and goals; communication with HCP, easy to use; useful for newly diagnosed gestational diabetic mums; interest maintained till advanced stage pregnancy; customisable food and activity recommendation, interactive communication function requested; Unnecessary hobby questions; clinical look app preferred; general pregnancy information needed; more features better to maintain interest | Good |
| 3 | Scheibe M et al 2015 | Personal interviews | Germany | 32 participants  Type 2 Diab (63%) Type 1 (31%)  Hybrid (3%) Mean age 68.8 yrs | ON track Diabetes (Medivo) Glukose Monitor version 2.7, Diabetes plus 34% highly interested in tech 53% open minded 13% no interest in tech Interviewed 8 above 50 yrs old 25% owned Smart phone 47% knew apps 6% use apps | App handling issues; financial burden; lack of interoperability function; unintuitive functions and symbols; small fonds; no room for remarks along with BGM; reminders for medication and BGM, reference for diabetes knowledge, requested for nutritional facts on meals outside home; lacking individualised features | Good |
| 4 | Burgermaster, M et al 2023 | semi structured indepth interviews | USA  (Northern Manhattan) | 35 participants Type 2 diabetes Hispanics (Under served)  18-65 yrs | Platano app. | Easy to use, internal motivation: lead healthy lifestyle; social support from HCP and peers to keep them in check; health literacy improved | Good |
| 5 | Steinman L  et al 2020 | interview focus group discussion | Cambodia | 70 participants  diabetes & hypertension (73%) diabetes (70%),  hypertension (75%).  Mean age 56 years | No info on app or non app users All owned Smart Phones | Reminders for medication, lab tests and clinic visits; improved knowledge on disease, food choices and physical activity; preferred voice over text; social support needed such as family for technical support | Good |
| 6 | Torbjonssen A  et al 2019 | Semi structured in-depth interview telephone or face to face | Europe (Norway) | 24 participants  Type 2 Diabetes Av age 61 yrs, | Diabetic diary app  1 year of use | Easy to use, good visual presentations of BGM, diet and exercise; organised feature of glucose uploading; able to understand relationship between causality and glucose levels; Dietary restriction difficult; auto tracking of BGM recommended, HCP connection feature recommended for better glucose control; motivate habit formation; app helped make self- management decision; unable to interpret data; technical difficulties | Good |
| 7 | Desveux L.  et al 2018 | semi structured one to  one interview by telephone | Canada | 26 participants Type 2 Diabetes | WelDoc Bluestar app Participants instructed to use the app for 3 mths, followed by interview | Increased awareness of disease management; motivating; feedback enhances positive behavior; time consuming effort; technology not perceived as advantage | Fair |
| 8 | Alkalwadeh M  et al 2020 | Semi structured interview | USA (Western  Massachusetts | 12 participants Type 2 diabetes Black and White Latino/ Hispanics 55yrs and above | ASSISTwell app on Tablet  30 days usage | Sense of accountability; self-dependence; stay on track; instill discipline; useful reminders; sense of control; source of support sharing with HCP; high level of acceptance and trust; become more compliant; positive change in behaviour; perceived as a tool in facilitating the self-management tasks | Good |
| 9 | Cheng et al 2022 | semi structured interview | Shanghai | 20 participants Type 2 Diabetes  average age 60 yrs | WECHAT based mHealth  Current and non- users of app | Functions are practical; poor understanding of features fonts too small; request for more audio and visual aids; must be easy to use; strong need for security; healthy food options besides nutritive value only; real time monitoring of blood glucose; need modification on monitoring functions for older folks; want latest info on diabetes medication; connection with HCP, and TCM guidance features to analyse BGM result; social influence to help use; credible source of info required; face to face assistance for initial instruction to use app | Good |
| 10 | Lewinsky A et al 2021 | Semi structured interview by telephone | South Eastern  United States | 20 participants  Type 2 Diabetes  blacks and Whites.  Mean age 57yrs | FITBIT ATLA S Fitness tracker, IHealth glucometer,  Body trace SCALE.  Use for 6 mths | Easy to use for all 3 apps; tech issues with; Blutooth connection, log in, updating BGM issues and inconsistent cellular connectivity; reminder features was useful; engage them in self- management routine; request for HCP data sharing facilility; useful data visualization; uncomprehend able variation in blood glucose readings linking to health behaviour; detailed information required on blood glucose reading such as to allow longer monitoring time and ability to predict HBA1c value and av blood glucose levels over time | Fair |
| 11 | Franklin R  et al 2019 | Semi structured interview face to face | UK | 8 participants Type 1 diabetes age between 27-57 yrs | current and non- current users of app | Integration of blood pressure readings preferred; HBA1c, exercise, food intake and insulin dose in graphical format to understand their relationships; visual patterns of blood glucose level for different times of day; incorporate colour scheme for different levels of blood glucose levels; easy to use; HCP data sharing facility requested | Good |
| 12 | Kwan et al 2023 | Semi structured interview | Singapore | 9 participants Type 2 Diabetes 40-69 yrs of age | EMPOWER app at point of interview  10 mins familiarising the app | Useful reminder notifications; Bluetooth integration for glucose and blood pressure readings for ease of data entry; large food database with sugar content; one touch login of medication and favourite food; ability to share data with HCP | Good |
| 13 | Yoon et al 2022 | Focus group discussion one to one interview | Singapore | 29 participants  type 2 Diabetes mean age 57.8 yrs old | Present features in existing app | Quantifiable goal setting; personalised nudges based on individual tracked data; resources from credible sources; social interaction with peers and HCP | Good |
| 14 | Fu et al 2023 | electronic Survey responses  with app testing | USA | 92 participants type 1 and 2 diabetes whites Mean age: 54 years | electronic Survey responses  with app testing | Competence, autonomy and connectivity importance; competence: help track BGM and explain the levels, autonomy: help set personalised behavioral goals based on individual day and time of week, connectivity : share data records and communicate with others for self-management advice; request for auto data upload; alert, reminder, voice recognition preferred | Fair |
| 15 | Klemme I et al 2023 | semi structured interview | Germany | 24 participants  Type 1 diabetes  23-81 yrs | current Diabetic app user  some without app experience | Requested sharing information capabilities; user friendly; reliable sources of information; requested for discreet messaging option via Smartwatches; personalised information lacking; poor sensors and app connectivity; direct communications with HCP lacking; customised alarm tone to minimize stigmatization; security issue resulted in hampering sharing data with healthcare system | Good |
| 16 | Luo et al 2021 (under privileged) | semi structured interviews | USA | 15 participants   Type 1 , Type 2 diabetes adults > 18yrs | Diabetes app | To individualise patients' needs; technical and security challenges; willing to try app | Good |
| 17 | Leziak K et al 2021 | Focus group, semi  structured interviews | USA (Chicago) | 37 participants gestational Diabetes  low income | Use of diabetes app Non-current user | Useful glucose tracking and food login features; interactive features; one stop information such as feedback on food choices and recipes requested; real time communication with HCP preferred; monitoring task reminders favoured | Good |
| 18 | Jeffery B et al 2019 | Interview | Australia | 30 participants Type 2 Diabetes mean age 60-69 yrs | current and non app user  ACCUCHECK, Diabetes Journal  food and physical tracking app | Connectivity issues; misconception on diabetes severity therefore underestimated value of app; unaware of existence of diabetes app; technical literacy issue; useful BGM and food intake tracking; non- user open to using app; motivated to use if HCP recommends; satisfied with current GP treatment without app assistance | Good |
| 19 | Ritholz M et al 2019 | In-depth interview | USA (Boston) | 10 participants Type 1 Diab Mean age 52 years | Sugar Sleuth app  1 year of usage | Self-empowering, developed self-monitoring habits, improve dialogue with clinicians; perceived as psychological support; positive sentiments on self- management; excessive information demanded; not suited for the less privileged | Good |
| 20 | Kabeza CB et al 2020 | Semi structured face to face in depth interview | Central Africa | 14 participants Type 1 or 2 Diabetes F 6 /8 male Mean age 36 yrs | Kir' App  3 mths of usage | Satisfied with the knowledge received for newly diagnosed; happy with the forum provided on individual experience with diabetes; local services information on pharmacy whereabouts needed; useful monitoring and reminder functions; helpful BGM visual trend graphs; good information on diet and exercise; motivational messages requested; feel empowered; facilitated shared clinical decision making with GPs; psychological support; awareness of their conditions; lack of audio or video functions perceived as setbacks; lack of social connection features | Good |
| 21 | Rossmann C  et al 2019 | Semi structured face to face interviews (Both studies) | Study 1: Singapore  Study 2: Germany  (Munich) | Study 1  21 participants  Type 1 and 2 (SG) Age: 29-64 yrs Study 2 16 participants Type 1& 2 Diabetes Age :29-64 yrs | Singapore & Germany: GlyCO App, MySugr Academy  Fee Style LIBRE, DEXCOMM, ACCU Check Type 1- app users Type 2- only some are current app users usage range from few days to years | Singapore Study 1: favoured glucose monitoring features, Type 1 favoured the nutrition support; Cost, time and tech issues; lack of chat box with organization; unsupportive HCP; German study 2: useful lifestyle and physical trackers | Good |
| 22 | Kelly L et al 2018 | In depth interviews | UK | 15 participants Type 2 Diabetes Av age 55.4 yrs | current user of app with diabetes self management | Understanding individual health; goal fulfillment; reduce disruption to daily routine; psychological support; interaction with HCP; recommended to be part of integrated care | Good |
| 23 | Knight BA et al 2016 | Focus Group Discussion | Australia | 7 participants Type 1 Diabetes mean age 36 yrs, | Rapidcalc app 1 mth usage | Trust Insulin bolus calculation; convenient record of data and information; convenient; helps to reduce risk of hypoglycaemia | Good |
| 24 | Peng W et al 2015 | Focus Group Discussion | USA (Mid west) | 18 participants Type 2 Diabetes Average age: 54 yrs | Glucose Buddy App MYSugr MYFitnessPal, MapMyWalk apps  1 hr daily for 22 mths | Task monitoring feature favoured; lack of recommendation from HCP; customised feedback recommended; credible source of information; attention grabbing reminders recommended; no room for home food input; small achievable goal-setting feature preferred; sync data with hospital health record facility required; to allow data sharing with friends for support | Good |

## Appendix 3 – CASP Summary Table

| **AUTHOR** | **Was there a clear statement of the aim of the research?** | **Is qualitative methodology appropriate?** | **Was the research design appropriate to address the aims of the research?** | **Was the recruitment strategy appropriate to the aim of the research?** | **Was the data collected in a way that address the research question?** | **Has the relationship between researcher and participant been adequately considered?** | **Have ethical issues been taken into consideration?** | **Was the data analysis sufficiently rigorous?** | **Is there a clear statement of findings?** | **How valuable is the research?** |
| --- | --- | --- | --- | --- | --- | --- | --- | --- | --- | --- |
| **Alkawadeh MY et al 2020** |  |  |  |  |  |  |  |  |  |  |
| **Bults M et al 2023** |  |  |  |  |  |  |  |  |  |  |
| **Burgermaster M et al 2023** |  |  |  |  |  |  |  |  |  |  |
| **Cheng K. et al 2022** |  |  |  |  |  |  |  |  |  |  |
| **Desveux L et al 2018** |  |  |  |  |  |  |  |  |  |  |
| **Franklin R et al 2018** |  |  |  |  |  |  |  |  |  |  |
| **Fu HNC et al 2023** |  |  |  |  |  |  |  |  |  |  |
| **Jeffery B et al 2019** |  |  |  |  |  |  |  |  |  |  |
| **Kabeza CB et al 2020** |  |  |  |  |  |  |  |  |  |  |
| **Kelly L et al 2018** |  |  |  |  |  |  |  |  |  |  |
| **Klemme I et al 2023** |  |  |  |  |  |  |  |  |  |  |
| **Knight BA et al 2016** |  |  |  |  |  |  |  |  |  |  |
| **Kwan YH et al 2023** |  |  |  |  |  |  |  |  |  |  |
| **Kyto M et al 2022** |  |  |  |  |  |  |  |  |  |  |
| **Lewinsky A et al 2021** |  |  |  |  |  |  |  |  |  |  |
| **Leziak K et al 2021** |  |  |  |  |  |  |  |  |  |  |
| **Luo J et al 2023** |  |  |  |  |  |  |  |  |  |  |
| **Peng W et al 2015** |  |  |  |  |  |  |  |  |  |  |
| **Ritholz M et al 2019** |  |  |  |  |  |  |  |  |  |  |
| **Rossmann C et al 2019** |  |  |  |  |  |  |  |  |  |  |
| **Scheibe et al 2015** |  |  |  |  |  |  |  |  |  |  |
| **Steinman L. et al 2020** |  |  |  |  |  |  |  |  |  |  |
| **Torbjonssen A. et 2019** |  |  |  |  |  |  |  |  |  |  |
| **Yoon S et al 2022** |  |  |  |  |  |  |  |  |  |  |

|  | **Yes** |
| --- | --- |
|  | **Can't tell** |
|  | **No** |
|  | **Very valuable** |

## Appendix 4 – Data synthesis of twenty-four studies

|  | Author |  |
| --- | --- | --- |
| 1 | **Kyoyto M et al 2022; Gestational Diab** | well-being \| feedback progress \| tailored nutrition/ exercise suggestion \| eating reminder \| glucose walk effects \| sleep data input \| routine habits input \| personal greeting \| partners' app version \| social support group \| general preg info \| self-tracking glucose \| integrate with HCP \| more features \| tolerate usage breaks |
| 2 | **Bults M 2023; Type 2 Diabetes** | healthy lifestyle monitoring \| continuous monitoring glucose \| logging excessive info \| assistance required \| friends/relatives comments important \| HCP approved apps \| insurance reimbursement |
| 3 | **Scheibe M 2015; Diabetes (1 & 2 Diab)** | complicated \| time consuming \| expensive \| privacy issue \| lack of interoperab \| Identifying touch screen difficulty \| difficulty switching numbers and letters \| small font size \| Needs not tailored \| Missing polypharmacy \| management \| personal text on glucose levels \| colour codes glucose levels \| Nutritional info restaurant meals \| one page monitoring tasks \| medication reminder useful \| reference for diabetes info |
| 4 | **Burgermaster M et al 2023 (Type 2)** | 8/35 tech difficulties \| 28/35 easy to use \| computing literacy deficit \| positive experience \| motivating \| sense of autonomy \| blood glucose & meal tracking \| increase self awareness \| improve attitude self management \| increase knowledge \| lack of accountability |
| 5 | **Steinman L et al 2020; Unspecified Diabetes** | phone literacy issue \| Voice messages preferred \| small font \| message simple and fun \| frequent health messages \| motivating, dev skills, informative \| reminder tool for all tasks \| money /time issue- does not solve problem (med) |
| 6 | **Torbjornsen et al 2019; Type 2 Diabetes** | inspiring to self manage \| Easy accessibility \| automatic transmission data \| Provide structure organised \| visually appealing data \| complicated, time consuming \| blood glucose data transfer issue \| stressful constant reminders \| poor health hamper app usage \| small buttons failure \| costly data outside home \| Automatic tracking \| Flexibility across devices \| Interconnection with HCP \| meaningful routine \| good explanation of result \| inteprating data shortcomings \| dietay registration difficulty \| insufficient edu support \| require detailed feedback \| receive at early stage \| unsupportive HCP |
| 7 | **Desveaux L et al 2018; Type 2 Diabetes** | visually appealing data \| motivating : graphs presentation \| increase awareness \| useful glucose/diet tracking (2nd grp) \| enhanced accountability \| performance feedback enhance positive behaviour \| Increase confidence \| Time consuming (3rd Group) \| preferred log book method |
| 8 | **Alkawaldeh MY eta al ; 2020 (Type 2 Diabetes)** | enhanced self dependence \| enhanced happiness satisfaction \| enhanced timely monitoring \| push to monitor \| blood glucose tracking beneficial \| enhanced accountability \| keep on their toes \| increase awareness \| positive behaviour/attitude \| enhanced confidence \| beneficial reminders \| sense of support \| Data recording shared with HCP \| high level of trust/ acceptance \| more engaged in diabetes management |
| 9 | **Cheng K. et al 2022; Type 2 Diabetes** | font size too small \| difficult medical terms \| illustrations /audio explanations needed \| design not intuitive \| security concern \| complimentary preferred or small fee \| knowledge on diet, exercise, blood sugar \| food replacement suggestion \| dietary scientific information \| range of exercises provided \| guidance on medication \| updated medication development \| TCM complimenting option \| reminders \| statistical analysis of health indicators \| linked with HCP \| means of healthcare access \| family assistance in app navigation \| peer influence \| credible information source |
| 10 | **Lewinsky AA et al 2021; Type 2 Diabetes** | easy and feaseable \| easy physical tracker \| complicated glucose tracker steps \| connectivity issue \| cellular connectivity physical tracker issue \| happy with step recording \| positive health behaviour \| failitated dialogue with HCP \| lack of glucose level variation explanation \| Visual data enhances self monitoring \| average blood glucose levels \| Longer glucose data timeline \| Predictive HBA1c level |
| 11 | **Franklin R et al 2018; Type 1 Diabetes** | good visualisation of health indicator data \| data integration of exercise, diet and glucose levels \| identify blood glucose trend \| blood glucose reading in colour \| blood glucose reading insulin, carbohydrate intake in line graphs \| easy to use, convenient \| personalised design \| linked with HCP to share data |
| 12 | **Kwan YH et al 2023; Type 2 Diabetes** | backdating of physical activity logging \| textbox for glucose reading, options for mmol and dcl units \| Integrate with glucometer \| allow food type and variable portions consumed entry \| healthy food guidleines \| Flexible medication entry \| manual customised medication input \| synchronise with fitness trackers \| medication reminders \| medical appointment reminder \| blood glucose reading alerts to HCP \| incorporate educational games \| rewards eg vouchers, glucose strips dsicount \| chat feature with HCP and other users \| diabetes education & treatment update |
| 13 | **Yoon S et al 2022; Type 2 Diabetes** | reminders \| blood glucose tracking \| personalised guidance on diet/ exercise \| time consuming data loggin \| healthy lifestyle knowledge \| credible accurate information \| app based peer support \| chat box with HCP |
| 14 | **Fu HNC et al 2023** | improve competence \| create analysis report \| counting carbohydrate function \| blood sugar tracking for insulin adjustment \| data visulaisation enhances self empowerment \| assist in behavior change decision making \| beneficial bidirectional messaging function with HCP \| coaching support \| efficient and easy \| patient friendly terminology \| interoperable between devices \| voice recognition \| reminders \| app ineteractivity for behaviour confirmation \| visually appealing, colours, icons, emojis, fun \| connect to pharmacy |
| 15 | **Luo J et al 2021; Type 1 or 2 Diabetes** | individualised app function in medication adherence and dietary needs \| reminders \| incorporate diabetes friendly shopping list \| easy to use \| avoid medical jargons \| combination of glucose and diet tracker \| privacy and security issues |
| 16 | **Leziak K et al 2021; Gestational Diabetes** | glucose level tracking \| Dietary input \| monitoring reminders \| reminders for action on complex tasks \| physical and weight tracking \| feedback on food choices recipes \| Real time communciation with HCP |
| 17 | **Jeffery B et al 2019; Type 2 Diabetes** | technical difficulties apps crashing \| uncustomised measurement units \| unintuitive navigation \| cost issue \| small font size \| unsupportive HCP \| user friendly \| beneficial visual glucose representation \| synchronisation with glucometer \| credibility and non personalised information \| lack of trend/ averaging glucose monitoring \| inability to associate BGR trend with food intake \| no carbohydrate content calculation \| no one stop BGR storage \| use app upon HCP recommendation |
| 18 | **Ritholz MD et al 2019; Type 1 Diabetes** | self empowerment tool \| promoted pre emptive self care actions \| feedback for corrective actions \| increases understanding of healthy food choices \| actively engaged with HCP in management \| ease burden on self monitoring tasks \| confident in effective managment of glucose level \| beneficial graphic representation \| long term and short term glycaemic data choices \| mismatched information demanded |
| 19 | **Kabeza CB et al 2020; Type 1 or 2** | filled knowledge gaps \| individualised corrective action \| forum for queries \| local services information \| dispel diabetes myths \| monitoring reminders \| informed food choices \| variety of exercises & intensity recommendations \| help cope with emotional stress \| absence of social support \| enabled clnical shared decision making \| absence of audio/video \| appreciale local language availability \| knowledge enhancement over time |
| 20 | **Kelly L et al 2018; Type 2 Diabetes** | beneficial graphical representations \| longitudinal data identify trends \| alerts for corrective actions \| useful tracking function \| informative \| motivating \| gamification maintain engagement \| Continuous blood glucose sensors promotes early intervention \| nutritional content reference \| difficulties in data entry \| high reassurance on optimal self management \| point of discussion with HCP \| LInked with electronic medical record |
| 21 | **Knight BA et al 2016; Type 1 Diabetes** | ease of use \| trusted insulin bolus calculation \| CHO alert when blood glucose below target \| glucometer synchronise with app \| convenient record keeping \| web based data storage for safety \| favorite foods registration \| ability to associate blood glucose trend with food \| personalised screen displays |
| 22 | **Peng W et al 2015; Type 2 Diabetes** | technical literacy \| happy with paper and pen \| no recommendation from HCP \| disfavour behaviour accountability \| ease of use \| tasks monitoring \| customised feedback \| complexity of home made food entry \| attention grabbing reminders \| source of nutritional information \| non preachy style of information \| small achievable goal setting feature \| monetary rewards to maintain engagement \| open to sharing data with friends or \| synch self track data with hospital health record |
| 23 | **Klemmer I et al 2023; Type 1 Diabetes** | interoperable with hardware devices \| customised information \| communication with HCP and peers \| individualised alarm tones and time slots \| data security hampers data transferring to healthcare system |
| 24 | **Rossmann C et al 2019; Study 1 & 2 (Singapore & Germany); Mix of Type 1 and 2 Diabetes** | useful glucose and diet monitoring \| Automated glucose data transfer \| HCP recommendation dependent \| time consuming \| technical difficulties, crashing \| incompatiblity with glucometer \| facilitating tool \| chat with peers and organisation \| chat with HCP not neccesary \| fitness tracking |
