# Supplementary material for: What are the perceptions and experiences of adults using mobile applications for self-management in diabetes? A systematic review
Source: BMJ Open. 2025 Jan 20;15(1):e086671. doi: 10.1136/bmjopen-2024-086671 (PMC11751966; doi:10.1136/bmjopen-2024-086671)
Supplement: Supplementary file 2 [file bmjopen-2024-086671.reviewer_comments.pdf]

PEER REVIEW HISTORY

BMJ Open publishes all reviews undertaken for accepted manuscripts. Reviewers are asked to complete a checklist review form and are provided with free text boxes to elaborate on their assessment. These free text comments are reproduced below.

ARTICLE DETAILS

Title (Provisional)

What are the perceptions and experiences of adults using mobile applications for self-management in diabetes? A Systematic Review

Authors

Patail, Kalthum; Pai, Hui Hsuan; Greenfield, Geva; Hayhoe, Benedict; Majeed, Azeem; Neves, Ana Luisa; Taddese, Henock B

VERSION 1 - REVIEW

**Reviewer**	**1**
**Name**	**Baagar, Khaled**
**Affiliation**	**Hamad Medical Corporation**
**Date**	**18-May-2024**
**COI**	**None**

This is a systematic review to explore perceptions and experiences of adults with type 1,2 and gestational diabetes using mobile applications for self-management in diabetes.

I have this comment for the authors:

- Did you explore any assessment of application fatigue and loss of engagement with the apps?

**Reviewer**	**2**
**Name**	**Tsujii, Satoru**
**Affiliation**	**Tenri Hospital, Endocrinology**
**Date**	**16-Jul-2024**
**COI**	**None.**

The authors performed a literature search for the narrative studies that described the perceptions and experiences of using mobile applications in self-management of type 1, type

2 and gestational diabetes. Though their aim to explore the patient-reported usability of the apps that would help self-care people with diabetes was understandable, the concept and/or objective of each app seemed to be ambiguous, heterogenous or widely diverse. The common phrase was only mobile phone applications for people with diabetes and there was no description on QOL item for participants. The manuscript could make it a systematic review?

Because it must be certain that "Peer support is a source of motivation", people with diabetes might need technology like a personal AI which could listen to their needs and give some knowledge with some proposals. The authors could describe the feasible frame of the future applications.

VERSION 1 - AUTHOR RESPONSE

Reviewer 1: Did you explore any assessment of application fatigue and loss of engagement with the apps?	Thank you very much for your feedback. Asa systematic review, our synthesis was limited by the findings reported in the included primary studies.<br><br>Application fatigue and loss of engagement were not discussed directly in the included studies. However, the studies did highlight barriers to use that may influence engagement with the apps, such as frequent crashing of apps, slow downloading of information, interoperability issues such as incompatible monitoring device with app and Bluetooth connectivity issues. We have summarised and discussed these in our report. Please see pages 15 and 16 and 18 in the manuscript.
Reviewer 2: Though their aim to explore the patient-reported usability of the apps that would help self-care people with diabetes was understandable, the concept and/or objective of each app seemed to be ambiguous, heterogenous or widely diverse. No description on QOL items for participants. The manuscript could make it to systematic review?	Thank you for your feedback. As we focused on user's experiences that are captured through qualitative methods, we did not include QOL related synthesise.
Reviewer 2: Because it must be certain that "Peer support is a source of motivation", people with diabetes might need technology like a personal AI which could listen to their needs and give some knowledge with some proposals. The authors could describe the feasible frame of the future applications	Thank you for your feedback, we have included the following wording in our recommendations to emphasise the importance of peer support:<br><br>**Future Studies and policy implication:** Given that more than 90% of these identified studies consist of participants using the app for less than 6 months, it is difficult to predict the experience of persistent usage of the app in diabetes management and the role it will play in the entire healthcare system. The studies also revealed

	**'peer support' function facilitating exchange of ideas in managing diabetes is a source of motivation for patients. Hence, future studies that capture long term usage of mobile application incorporating personal AI in providing an interactive support that assists patients with self-management in the disease is recommended.**
